# Confirmation of the Absence of Somogyi Effect in Patients with Type 2 Diabetes by Retrospective Continuous Glucose Monitoring Systems

**DOI:** 10.1155/2022/6599379

**Published:** 2022-10-04

**Authors:** Yuxin Huang, Xudan Lou, Weicong Huang, Jieyuzhen Qiu, Cuiping Jiang, Jiao Sun, Xiaoming Tao

**Affiliations:** ^1^Department of Endocrinology, Huadong Hospital Affiliated to Fudan University, Shanghai 200040, China; ^2^Shanghai Zhengpu Technology Co., Ltd, Shanghai 200431, China

## Abstract

**Background:**

The Somogyi effect is defined as fasting hyperglycemia secondary to nocturnal hypoglycemia. In past decades, this effect proved to be rare or absent. However, many endocrinologists still believe in this phenomenon in clinical practice. Does the Somogyi effect truly exist? We aimed to answer this question with a study based on a larger sample size.

**Methods:**

We collected retrospective CGMs data from 2,600 patients with type 2 diabetes with stable treatment of insulin. Nocturnal hypoglycemia was defined as a CGMs sensor glucose of less than 3.9 mmol/L for at least 15 min between 24:00 and 06:00. Morning fasting glucose was compared between people with nocturnal hypoglycemia and without nocturnal hypoglycemia.

**Results:**

Valid CGMs data were obtained on 4,705 of 5,200 nights. Morning fasting glucose was observed lower after nights with nocturnal hypoglycemia compared with nights without hypoglycemia (*P* < 0.001). 84 cases presented fasting glucose of more than 7 mmol/L after nocturnal glucose of less than 3.9 mmol/L. Only 27 cases presented fasting glucose of more than 7 mmol/L after nocturnal glucose of less than 3.0 mmol/L. Fasting glucose values below 3.9 mmol/l in the morning were associated with a 100% risk of nocturnal hypoglycemia, while fasting glucose values over 9.6 mmol/l in the morning were associated with no risk of nocturnal hypoglycemia. Correlation analysis showed that the nocturnal glucose nadir was significantly correlated with fasting glucose levels (*r* = 0.613, *P* < 0.001).

**Conclusions:**

Our data provided no support for the existence of the Somogyi effect. If fasting glucose exceeds 9.6 mmol/L, we do not have to worry about asymptomatic nocturnal hypoglycemia in patients with type 2 diabetes.

## 1. Introduction

It was first reported by Michael Somogyi in 1959 that nocturnal hypoglycemia may lead to fasting hyperglycemia the next morning, which is known as the “Somogyi effect” [1] or “rebound hyperglycemia.” It is a famous hypothesis in the management of diabetes. If the “Somogyi effect” does exist, it means clinicians need to increase or decrease insulin doses based on nocturnal glucose levels, not just fasting glucose levels.

Although several studies have supported the existence and pathogenesis of Somogyi effect [2–4] which has been written into textbooks, most published evidence does not support the hypothesis [5–7]. With the advent of continuous glucose monitoring systems (CGMs), the Somogyi effect proved to be rare or absent in most studies in the following decades [8–10]. The fasting glucose levels after nights with nocturnal hypoglycemia were lower than those after nights without nocturnal hypoglycemia, and the more severe the nocturnal hypoglycemia was, the lower the fasting glucose levels were. However, the sample sizes of these studies were not large. A few patients still presented fasting glucose of more than 10 mmol/L despite nocturnal hypoglycemia in these studies. In clinical practice, many endocrinologists still believe in this phenomenon [11, 12]. Does the Somogyi effect truly exist? We aimed to answer this question with a study based on a larger sample size.

## 2. Materials and Methods

This is a retrospective study. We collected retrospective CGMs (Medtronic MiniMed CGMS system gold™ and iPro™ 2 Professional CGMs, Medtronic Inc., USA) data from 2,600 patients with type 2 diabetes in the department of endocrinology, Huadong Hospital affiliated to Fudan University, from May 2010 to September 2021. The study protocol was approved by the Ethics Committee of Huadong Hospital. All procedures performed in studies involving human participants were in accordance with the ethical standards of the institutional research council and the Declaration of Helsinki. All subjects gave their informed consent.

The inclusion criteria were as follows: (a) diagnosis according to the 1999 World Health Organization criteria; (b) stable treatment with insulin combined with or without oral hypoglycemic agents for at least four weeks; and (c) HbA1c of less than 9%. The exclusion criteria included the following: (a) current diabetic ketoacidosis or hyperosmolar coma; (b) current cardiovascular disease or other serious disease; (c) severe hepatic or renal insufficiency; (d) symptomatic hypoglycemia recorded during CGMs; (e) eat snacks or treat a hypoglycemia that occurred during the CGMs nights; and (f) incomplete glucose monitoring data.

The CGMs sensor was installed on day 0 and removed on day 3. The CGMs data on day 1 and day 2 were obtained to avoid any interference due to the installation and removal of sensor. Nocturnal hypoglycemia level 1 was defined as the CGMs sensor glucose of less than 3.9 mmol/L and greater than or equal to 3.0 mmol/L for at least 15 min between 24:00 and 06:00, and nocturnal hypoglycemia level 2 was defined as the CGMs sensor glucose of less than 3.0 mmol/L for at least 15 min between 24:00 and 06:00 [13–15].

Statistical analyses were performed with SPSS version 23.0 software (SPSS Inc., Chicago, IL, USA). Continuous variables were expressed as mean ± SD and were analyzed using ANOVA and post hoc analysis. Categorical variables were expressed as numbers (%) and analyzed by using the chi-squared test. Relationships between fasting glucose and nocturnal nadir glucose were calculated using Pearson's coefficient. A *P* value of <0.05 was considered statistically significant. The figures were created using GraphPad Prism 5 (GraphPad Software Inc., San Diego, CA, USA).

## 3. Results

In total, 2,600 patients with type 2 diabetes were enrolled in our study. Valid CGMs data on 4,705 (90.5%) of 5,200 nights were obtained, including 58% males and 42% females, with a mean age of 63.7 ± 12.7 yr, mean body mass index (BMI) of 24.8 ± 3.5 kg/m^2^, mean fasting glucose of 7.4 ± 1.5 mmol/L, and mean HbA1c level of 7.6 ± 1.1%. 79.5% were treated with oral hypoglycemic agents and insulin, and the rest were treated only with insulin. The median insulin dose was 18U per day. 6.6% were treated with neutral protamine Hagedorn (NPH) insulin; 39.9% were treated with premixed insulin, and 53.5% were treated with basal insulin analogs.

**Figure 1:**
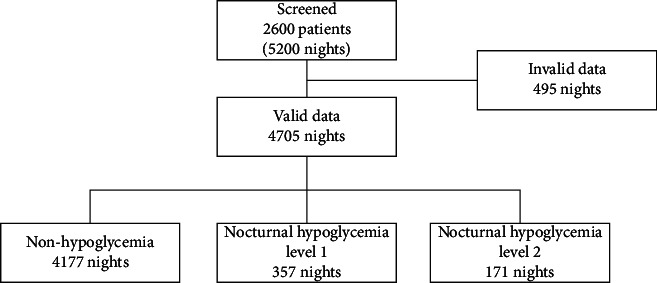
Patients' disposition.

According to nocturnal nadir glucose, these valid data were divided into three groups: non-hypoglycemia (4,177 nights), nocturnal hypoglycemia level 1 (357 nights), and nocturnal hypoglycemia level 2 (171 nights). There was no difference in the age, gender, BMI, HbA1c, renal function, oral hypoglycemic agents, and type of insulin among the three groups. Patients with nocturnal hypoglycemia level 2 presented longer diabetes duration, lower C peptide, and higher insulin dose than those without hypoglycemia and those with hypoglycemia level 1 (*P* < 0.05). Compared with fasting glucose levels after nights without hypoglycemia, lower fasting glucose levels were observed after nocturnal hypoglycemia level 1 and nocturnal hypoglycemia level 2 (*P* < 0.001) (see Figure 1 and Table 1).

84 cases (23.5%) presented fasting glucose of more than 7 mmol/L after nocturnal hypoglycemia level 1. Only 27 cases (15.8%) presented fasting glucose of more than 7 mmol/L after nocturnal hypoglycemia level 2. In these 27 cases, no one was treated with NPH; 15 cases (55.6%) were treated with premixed insulin and 12 cases (44.5%) were treated with basal insulin analogs. These 27 cases presented longer diabetes duration and older age than other cases in the group of nocturnal hypoglycemia level 2 (*P* < 0.001). No capillary blood glucose of less than 6 mmol/L or snacking was recorded at bedtime. By contrast, only 9 cases (0.2%) presented fasting glucose of less than 4.4 mmol/L after nights without hypoglycemia.

**Figure 2:**
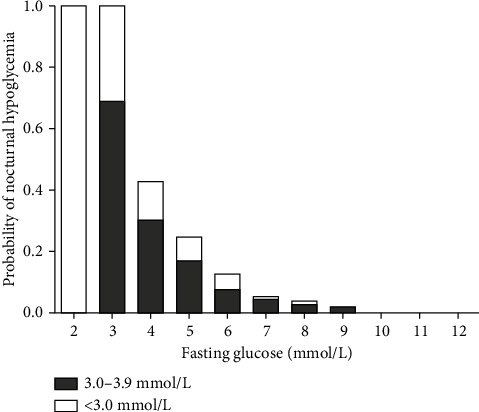
Risk of nocturnal hypoglycemia according to morning fasting glucose in 4,705 nights. Black bars, nocturnal hypoglycemia level 1 nights; white bars, nocturnal hypoglycemia level 2 nights.

**Figure 3:**
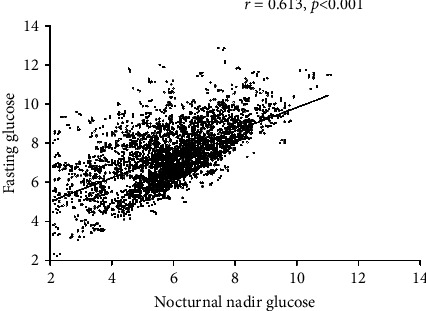
Relationship between nocturnal glucose nadir and morning fasting glucose levels.

Figure 2 presents the relationship between fasting glucose levels in the morning and the risk of nocturnal hypoglycemia in 4,705 nights. Fasting glucose values below 3.9 mmol/L in the morning were associated with a 100% risk of nocturnal hypoglycemia, while fasting glucose values over 9.6 mmol/L in the morning were associated with no risk of nocturnal hypoglycemia. Correlation analysis showed that the nocturnal glucose nadir was significantly correlated with fasting glucose levels (*r* = 0.613, *P* < 0.001, Figure 3).

## 4. Discussion

It is generally believed that fasting hyperglycemia in people with diabetes may be caused by the dawn phenomenon or the “Somogyi effect” [12]. The dawn phenomenon is defined as a spontaneous increase in plasma glucose toward the end of the nocturnal period in the absence of nocturnal hypoglycemia or any intake of dietary carbohydrates. The magnitude of the dawn phenomenon was calculated as the difference between the nocturnal nadir and fasting glucose level at a threshold of 20 mg/dL (1.1 mmol/L) [16]. In a previous study, this phenomenon could not be eliminated by any of the currently available oral hypoglycemic agents [11]. The use of basal insulin may be the best method to control the dawn phenomenon [17]. However, it is believed that the “Somogyi effect” is due to the release of counter-regulatory hormones (such as epinephrine, growth hormone, and cortisol) in response to nocturnal hypoglycemia induced by excessive amounts of exogenous insulin [2]. Therefore, the dawn phenomenon and Somogyi effect should be treated in an opposite manner. In clinical practice, clinicians often require patients with uncontrollable fasting hyperglycemia to undergo nocturnal glucose monitoring or CGMs. Hypoglycemia was proved to be associated with mortality in participants in both the standard and the intensive glycemic arms of the ACCORD trial [18]. If there is nocturnal hypoglycemia, the dose of insulin needs to be reduced; otherwise, the dose of insulin needs to be increased.

Although the existence of the dawn phenomenon is indisputable, the Somogyi effect remains a matter of debate. The Somogyi effect, if present, implies a robust counter-regulatory response that overcomes the hypoglycemic effect of insulin and raises the blood glucose levels above normal in the morning. In two recent studies, researchers have put forward strong evidence to challenge the Somogyi effect using CGMs [8,9]. In these studies, the mean fasting glucose levels after nights with hypoglycemia were significantly lower than those after nights without hypoglycemia in different people. Fasting glucose of less than 5 mmol/L indicates a high probability of asymptomatic nocturnal hypoglycemia. Hirsch et al. designed an interesting study to test the hypothesis that nocturnal hypoglycemia led to hyperglycemia the next day in 10 patients with insulin-dependent diabetes mellitus [19]. Nocturnal hypoglycemia was prevented by intravenous infusion of glucose on one day and induced by intravenous insulin on another day. After the occurrence of nocturnal hypoglycemia, the levels of fasting glucose, morning glucose, afternoon glucose, and all-day glucose were not higher than those after the prevention of nocturnal hypoglycemia. Daytime glucose levels were unrelated to the concentrations of nocturnal plasma glucagon, epinephrine, norepinephrine, growth hormone, or cortisol. Another study confirmed similar results by Tordjman et al. [7]. Based on the above studies, nocturnal hypoglycemia should not be suspected when fasting glucose levels are high.

Although the above data suggest a direct relationship between nocturnal hypoglycemia and lower fasting glucose levels, the sample sizes of these studies were not large. Furthermore, they did not exclude the possibility that the “Somogyi effect” occurred occasionally. Choudhary et al. reported that on two occasions, fasting capillary blood glucose was more than 10 mmol/L after nocturnal hypoglycemia, while on both occasions, the bedtime capillary glucose of less than 3.5 mmol/L was recorded [8]. It suggested that these patients may have consumed additional carbohydrate. However, it still cannot explain why several patients presented fasting glucose of more than 7 mmol/L after nocturnal hypoglycemia.

In our study, we obtained similar results with a larger sample size from patients with diabetes receiving stable treatment of insulin. Compared with those after nights without hypoglycemia, lower morning fasting glucose levels after asymptomatic nocturnal hypoglycemia were observed. Correlation analysis showed that the nocturnal nadir glucose was significantly correlated with fasting glucose levels in the morning. Over 4,705 nights, fasting glucose values below 3.9 mmol/L in the morning were associated with a 100% risk of nocturnal hypoglycemia, while fasting glucose values over 9.6 mmol/L in the morning were associated with no risk of nocturnal hypoglycemia. Only 0.2% cases presented fasting glucose of less than 4.4 mmol/L after nights without hypoglycemia. Our data also showed that there is no evidence that high fasting glucose values in the morning indicate the occurrence of asymptomatic nocturnal hypoglycemia. The “Somogyi effect” is rare or absent. Interestingly, we can ensure that if fasting glucose exceeds 9.6 mmol/L, we do not have to worry about asymptomatic nocturnal hypoglycemia in people with type 2 diabetes. This may be useful in clinical practice.

There are still limitations in our study that need to be mentioned. As a retrospective study, we had not tested the release of counter-regulatory hormones (such as epinephrine, growth hormone, and cortisol) during the night. Furthermore, 27 cases presented fasting glucose of more than 7 mmol/L after the nocturnal glucose of less than 3 mmol/L in this study. Although there was no record of snacking or the capillary blood glucose of less than 6 mmol/L at bedtime, we cannot exclude that these patients may have consumed additional carbohydrate. We cannot provide an explanation for the glucose surge in those 27 cases, which could actually be the “Somogyi effect,” and which is why, the effect has never been completely disproved. Some researchers believed that hyperglycemia after nocturnal hypoglycemia was due to the lack of insulin rather than hormonal counter-regulation [9]. A carefully designed prospective study might clarify this question.

## 5. Conclusions

Our study yielded three interesting results: (a) our data provided no support for the existence of the Somogyi effect; (b) fasting glucose below 3.9 mmol/L in the morning was associated with a high risk of nocturnal hypoglycemia; and (c) if fasting glucose exceeds 9.6 mmol/L, we do not have to worry about asymptomatic nocturnal hypoglycemia.

## Figures and Tables

**Table 1:** Characteristics of participants illustrated by total population and by subgroups of nocturnal nadir glucose.

	Total	Nocturnal nadir glucose			*P* value
	Population (*n* = 4705)	≥3.9 mmol/L (*n* = 4177)	3.0–3.9 mmol/L (*n* = 357)	<3.0 mmol/L (*n* = 171)	
Age (y)	63.7 ± 12.7	63.7 ± 12.6	63.4 ± 12.9	64.6 ± 14.9	0.556
Male (%)	2729 (58)	2429 (58.2)	186 (52.1)	114 (66.7)	0.085
BMI (kg/m^2^)	24.8 ± 3.5	24.8 ± 3.5	24.6 ± 3.2	24.6 ± 3.4	0.507
Diabetes duration (months)	120 (48, 197)	120 (48, 192)	120 (36, 240)	180 (85, 242)	<0.001
HbA_1c_ (%)	7.6 ± 1.1	7.6 ± 1.1	7.5 ± 1.2	7.6 ± 1.1	0.181
Fasting glucose (mmol/L)	7.4 ± 1.5	7.6 ± 1.4	6.0 ± 1.4	6.0 ± 1.5	<0.001
<4.4 mmol/L	66 (1.4)	9 (0.2)	36 (10.1)	21 (12.3)	<0.001
4.4–7.0 mmol/L	1866 (39.7)	1506 (36.1)	237 (66.4)	123 (71.9)	
>7.0 mmol/L	2773 (58.9)	2662 (63.7)	84 (23.5)	27 (15.8)	
eGFR (ml/min)	93.9 (77.2, 112.1)	93.6 (77.2, 112.1)	96.9 (83.9, 112.6)	90.6 (72.7, 109.9)	0.669
Fasting C peptide (ng/ml)	1.7 (1, 2.5)	1.7 (1.1, 2.6)	1.6 (0.9, 2.2)	1.4 (0.8, 2.4)	0.011
Total insulin dose (U/d)	18 (9, 30)	18 (8, 30)	16 (9, 30)	22 (8, 36)	<0.001
NPH (%)	312 (6.6)	282 (6.8)	21 (5.9)	9 (5.3)	0.139
Premixed insulin (%)	1875 (39.9)	1683 (40.3)	120 (33.6)	72 (42.1)	
Basal insulin analogs (%)	2518 (53.5)	2212 (53)	216 (60.5)	90 (52.6)	

Data are expressed as mean ± SD or number (percentage) or median (interquartile range). BMI, body mass index; eGFR, estimated glomerular filtration rate; NPH, neutral protamine Hagedorn.

## Data Availability

The data used to support the findings of this study are available from the corresponding author upon request.
